# Vaccine nationalism and the dynamics and control of SARS-CoV-2

**DOI:** 10.1126/science.abj7364

**Published:** 2021-08-17

**Authors:** Caroline E. Wagner, Chadi M. Saad-Roy, Sinead E. Morris, Rachel E. Baker, Michael J. Mina, Jeremy Farrar, Edward C. Holmes, Oliver G. Pybus, Andrea L. Graham, Ezekiel J. Emanuel, Simon A. Levin, C. Jessica E. Metcalf, Bryan T. Grenfell

**Affiliations:** 1 Department of Bioengineering, McGill University, Montreal, QC H3A 0C3, Canada.; 2 Lewis-Sigler Institute for Integrative Genomics, Princeton University, Princeton, NJ 08540, USA.; 3 Department of Pathology and Cell Biology, Columbia University Medical Center, New York, NY 10032, USA.; 4 Department of Ecology and Evolutionary Biology, Princeton University, Princeton, NJ 08540, USA.; 5 Princeton High Meadows Environmental Institute, Princeton University, Princeton, NJ 08540, USA.; 6 Department of Epidemiology, Harvard T.H. Chan School of Public Health, Boston, MA 02115, USA.; 7 Department of Immunology and Infectious Diseases, Harvard T.H. Chan School of Public Health, Boston, MA 02115, USA.; 8 The Wellcome Trust, London, UK.; 9 Marie Bashir Institute for Infectious Diseases and Biosecurity, The University of Sydney, Sydney, NSW, Australia.; 10 School of Life and Environmental Sciences, The University of Sydney, Sydney, NSW, Australia.; 11 School of Medical Sciences, The University of Sydney, Sydney, NSW, Australia.; 12 Department of Zoology, University of Oxford, Oxford, UK.; 13 Department of Medical Ethics and Health Policy, Perelman School of Medicine, University of Pennsylvania, Philadelphia, PA 19104, USA.; 14 Princeton School of Public and International Affairs, Princeton University, Princeton, NJ 08540, USA.

## Abstract

Vaccines provide powerful tools to mitigate the enormous public health and economic costs that the ongoing SARS-CoV-2 pandemic continues to exert globally, yet vaccine distribution remains unequal among countries. To examine the potential epidemiological and evolutionary impacts of ‘vaccine nationalism’, we extend previous models to include simple scenarios of stockpiling between two regions. In general, when vaccines are widely available and the immunity they confer is robust, sharing doses minimizes total cases across regions. A number of subtleties arise when the populations and transmission rates in each region differ, depending on evolutionary assumptions and vaccine availability. When the waning of natural immunity contributes most to evolutionary potential, sustained transmission in low access regions results in an increased potential for antigenic evolution, which may result in the emergence of novel variants that affect epidemiological characteristics globally. Overall, our results stress the importance of rapid equitable vaccine distribution for global control of the pandemic.

The SARS-CoV-2 pandemic has led to more than 180 million infections and nearly 4 million fatalities to date (*1*). Effective vaccines [e.g., (*2*–*4*)] have now been approved and are actively being deployed, but numerous important questions remain. Eventually, community immunity may be attained through the deployment of vaccines; however if and when this occurs will be contingent on the characteristics of natural and vaccinal immunity (*5*–*7*). As illustrated by the rapid spread and high transmissibility of the ‘delta’ variant (*8*), SARS-CoV-2’s evolutionary potential is a major potential obstacle for control (*9*).

Due to strong public and political pressures and fear of waning immunity, some countries with high vaccine availability are currently resorting to ‘vaccine nationalism’: stockpiling vaccines to prioritize rapid access to their citizenry (*10*). Indeed, at the time of writing, 99 and 117 doses per 100 individuals have been administered in the United States and United Kingdom, respectively, while an average of 25 and 3.8 doses per 100 individuals have been administered in India and across Africa, respectively (*11*). Recently, the World Health Organization recognized that delayed access to vaccines in countries with low vaccine availability may lead to more evolutionary potential (*12*), which could result in immune escape or other phenotypic changes of interest (e.g., increases in transmission). The emergence of future variants capable of evading natural or vaccinal immune responses could threaten containment efforts globally. These concepts underlie the development of a number of policy tools, including the existing COVAX initiative. Furthermore, to ensure that vaccine distribution is ethically-sound and equitable, the “Fair Priority Model” has been proposed (*13*–*15*) as a potential replacement to the currently planned proportional allocation (by population size) from COVAX.

Prior work has explored optimal prophylactic vaccine allocation for minimizing the final epidemic size of a fully immunizing infection (i.e., one that can be modeled using a susceptible-infected-recovered (SIR) framework) (*16*); when interaction between communities (or countries) is considered, equal vaccine distribution is increasingly advantageous in terms of minimizing case numbers (*16*). Modeling studies have also shown that coordinated influenza vaccine sharing would reduce the financial and infection burden of influenza outbreaks globally (*17*). Similar problems related to optimizing vaccine allocation have also been explored in networks with community structure (*18*), as well as in the face of economic constraints (*19*) and vaccination coalition formation (*20*), and for SARS-CoV-2 with age- (and contact-) heterogeneity (*21*, *22*).

We have recently shown that the strength and duration of immunity elicited following infection or one or two doses of a vaccine will have a crucial impact on the medium-term epidemiological and potential evolutionary outcomes (*5*, *6*). Here we extend these analyses to address potential epidemiological and evolutionary consequences of policies of vaccine nationalism or equitable access for a range of assumptions regarding the robustness of host immune responses. In reality, vaccine distribution is a public goods problem (*23*), and the optimal “global” allocation projected based on evolutionary and immunological uncertainties may differ from national optima due to the actual economic landscape of each country. In all cases, every nation, however, has a shared interest in reducing the potential for novel strains to arise, achievable by minimizing the global infection burden.

We consider a trans-national extension of our model, comprising two countries with possibly different population sizes and seasonal transmission patterns. One country, the high access region (HAR), chooses to allocate a fraction *f* of the total vaccine supply to the low access region (LAR). The underlying immuno-epidemiological models for both countries account for both the duration of natural and vaccinal immunity and the residual decrease in host susceptibility to infection (relative to immunologically naive individuals) after full natural or vaccinal immunity has waned; these models are described in detail in (*5*, *6*), where the more detailed structure (*6*) accounts for immunity after one or two vaccine doses. In the first “decoupled” framework (top panel of Fig. 1), we assume that the epidemiological dynamics of both countries are entirely independent, with the exception of their respective vaccination rates; we also compute a measure for the global potential for viral evolution of immune escape (*6*). In the second “coupled” framework (bottom panel of Fig. 1), we allow for immigration of infected individuals between the countries at rate η (*19*). Additionally, we approximate the stochastic occurrence of potential transmission increases (PTIs) in each country: briefly, if the ‘potential net viral adaptation rate’ [see supplementary materials, fig. S3, and (*6*)] exceeds a threshold, then there is a nonzero probability that the transmission rate in both countries increases. This follows evidence of enhanced binding of the SARS-CoV-2 spike protein receptor binding domain (RBD) with the ACE2 receptor in more contagious SARS-CoV-2 variants, as well as potentially higher viral loads (*24*). Mutational changes to the spike protein furin cleavage site (e.g., at site 681) may also contribute to increased viral transmissibility (*25*). In this way, this assumption represents a pessimistic scenario where the evolution of pathogen immune escape is inevitably accompanied by increases in transmission, although we also compare our results with the more optimistic scenario where transmission increases do not occur (note that we are not modeling the complexities of variant dynamics and evolution explicitly). In reality, transmission increases may plateau (*26*), and viral evolution may have more subtle effects on disease transmission including modulating the susceptibility of partially immune hosts. The full mathematical details for both frameworks are described in the supplementary materials.

**Fig. 1:**
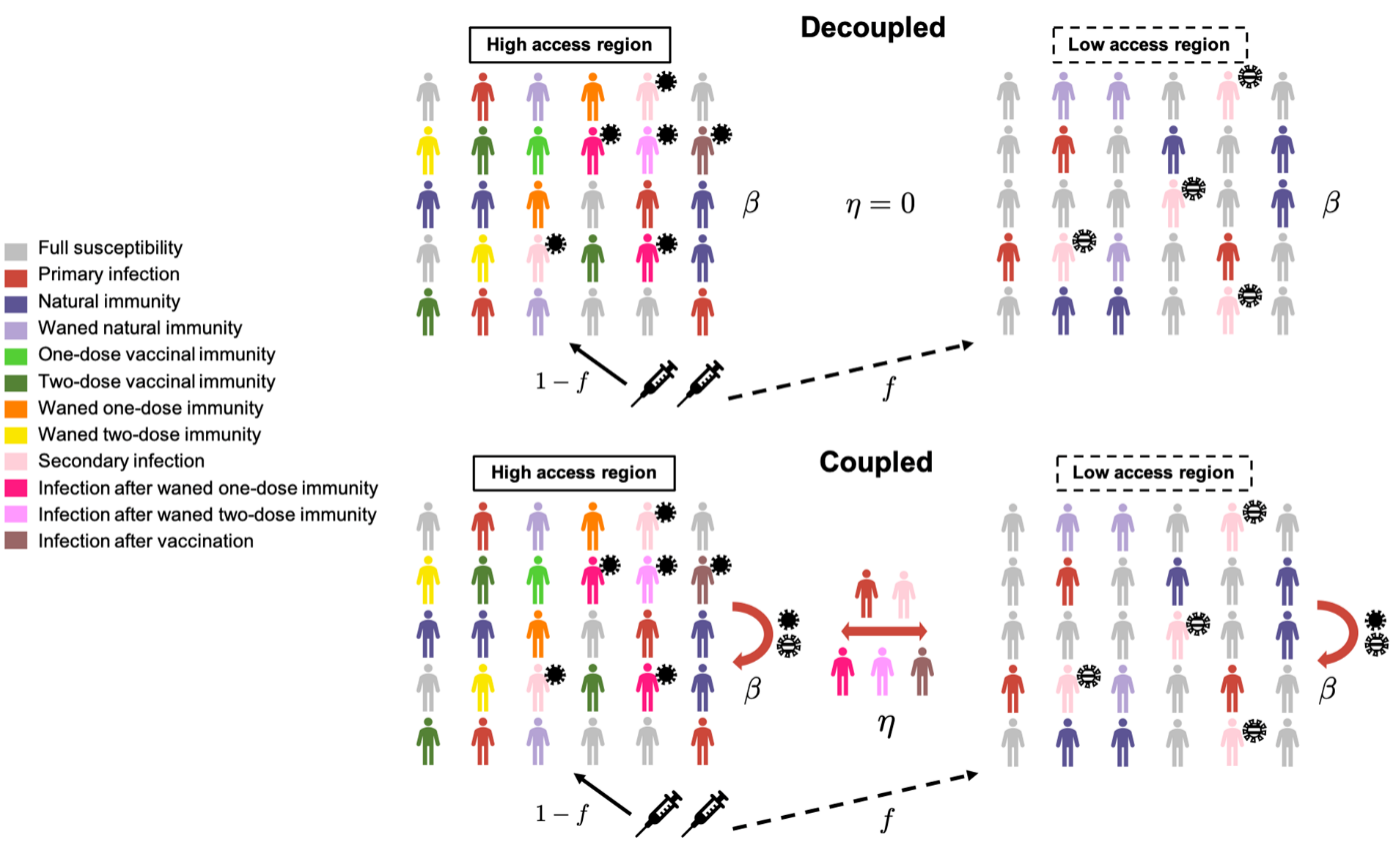
Schematic depicting the two-country model. The underlying immuno-epidemiological models for each country are based on (*5*, *6*). Vaccines are allocated by the high access region (HAR) to the low access region (LAR). In the coupled framework, immigration of infected individuals between the countries is considered, and the national transmission rate depends on potential transmission increases (PTIs) in both countries, shown schematically as solid and striped virus particles in the HAR and LAR, respectively. In the decoupled framework, no immigration occurs, and the transmission rate is not influenced by PTIs. Full model details are provided in the supplementary materials.

We begin with the decoupled framework and the simpler underlying ‘one-dose’ vaccination model from (*5*) to compute the long-term equilibrium fraction of infections in both countries under a range of epidemiological and immunological scenarios. Then, with specific dosing regimes (*6*), we examine the short- and medium-term epidemiological dynamics and the global potential for evolution with the sharing of vaccines. Next, using the coupled framework, we compute national and combined case numbers in the medium term given different degrees of vaccine allocation from the HAR to the LAR for different immigration rates and average relative reproduction numbers. We do so for total as well as severe cases, with the expectation that the number of severe cases may be indicative of infections requiring hospitalization or the clinical burden of Covid-19, while the number of total cases reflects all infections regardless of severity. Finally, we compare the results of the coupled and decoupled frameworks for specific scenarios.

## Decoupled framework

### Equilibrium analyses

**Fig. 2:**
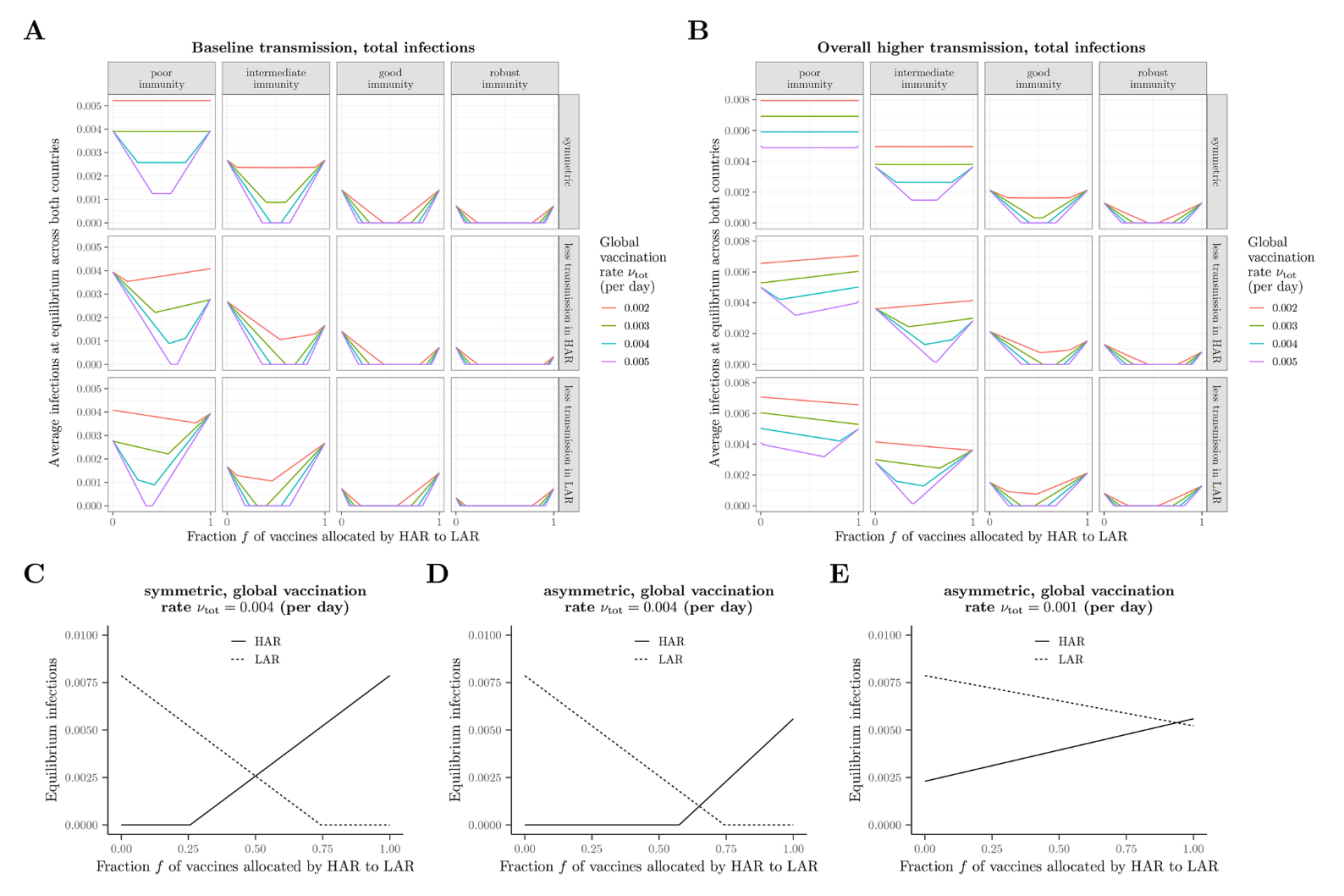
Long-term equilibrium of the average fraction of infections. (**A** to **E**) Equilibrium infections as a function of the vaccine fraction allocated by the HAR to the LAR under different scenarios related to immunity, transmission, and vaccination rate. In all panels, immunity scenarios are as follows: *poor immunity*, $\frac{1}{\delta }=\frac{1}{\delta_{\text{vax}}}=0.8\text{ years}$, ϵ=0.8; *intermediate immunity*, $\frac{1}{\delta }=\frac{1}{\delta_{\text{vax}}}=1\text{ year}$, ϵ=0.7; *good immunity*, $\frac{1}{\delta }=\frac{1}{\delta_{\text{vax}}}=1.5\text{ years}$, ϵ=0.6; *robust immunity*, $\frac{1}{\delta }=\frac{1}{\delta_{\text{vax}}}=2\text{ years}$, ϵ=0.5. In the scenario with asymmetrical transmission rates between the two countries, the transmission rate in the country with lower transmission is taken to be 80% of the value in the symmetric case. In the scenarios with overall higher transmission rates (B), this same asymmetric assumption is made in addition to the baseline symmetric transmission rate being elevated by 30% relative to the value in (A). In (C) to (E), illustrations of the equilibrium fraction of infections in each country with the intermediate immunity scenario are shown for (C) symmetric transmission with $\nu_{\text{tot}}=0.004$, (D) asymmetric transmission (lower in HAR) with $\nu_{\text{tot}}=0.004$, and (E) asymmetric transmission (lower in HAR) with $\nu_{\text{tot}}=0.001$, with all other parameters as in (A). In all panels, the baseline transmission rate is $\beta =\frac{2.3}{5}$.

To obtain analytical intuition for the effect of vaccine nationalism, we first examine the simplest model of vaccine sharing, where both regions are only coupled through their vaccination rates. The dynamics of prophylactic vaccine distribution strategies are well understood when infections lead to recovery and lifelong immune protection (SIR) (*16*). However, natural and vaccinal immunity to SARS-CoV-2 is likely not lifelong, yet complete re-susceptibility after the waning of immunity, as is assumed in susceptible-infected-recovered-susceptible (SIRS) frameworks, is also unlikely. Instead, we examine the role of both the strength and duration of immune responses with more general SIR(S) models (*5*, *6*). We first ignore the complexities of dosing regimes and extend the model in (*5*) to consider vaccine sharing in the decoupled framework (top panel of Fig. 1) assuming that a single immune category exists for vaccinated individuals, and that vaccinal immunity may wane at a rate distinct from natural immunity. Since we assume that the infection dynamics in both countries are only coupled through their respective vaccination rates, a unique equilibrium of total infections exists [either disease-free or endemic; see supplementary materials and (*5*) for details]. To examine the long-term epidemiological effects of vaccine nationalism, we compute the total fraction of infections at equilibrium as the proportion of vaccines shared between countries is varied. In other words, for a fixed global vaccination rate $\nu_{\text{tot}}$ (determined by the maximal rate of administration of the first dose $\nu_{0,\text{tot}}$ and the inter-dose period; see Materials and methods) and for a fraction *f* of vaccines allocated from the HAR to the LAR, the vaccination rates in the HAR and LAR are $\left(1-f\right)\nu_{\text{tot}}$ and $f\nu_{\text{tot}}$, respectively. We examine four immunity scenarios that range from poor to robust natural and vaccinal immune responses (Fig. 2).

When the characteristics of both countries are the same, sharing vaccines always decreases or maintains the total fraction of infections at the long-term equilibrium [see Keeling (*16*) for the SIR extreme with a focus on two differently-sized populations]. The intuition for this result is apparent from examining the underlying values for each country (Fig. 2C). The total fraction of infections are minimized whenever one of the countries does not vaccinate beyond the rate needed for herd immunity. Additionally, sharing does not have an appreciable impact on the total fraction of infections at equilibrium when vaccination rates are too low (Fig. 2A, top panel), or overall transmission rates are more elevated and host immune responses are poorer (Fig. 2B, top panel). Because of nonpharmaceutical interventions (NPIs) or intrinsic factors [e.g., demographics (*27*), population density (*28*), or vulnerabilities (*29*)], transmission rates in the two countries may be asymmetric. If there is less disease transmission in the HAR (modeled as a reduction in the transmission rate), then the ‘optimal’ fraction of vaccines shared to minimize the combined equilibrium fraction of infections crucially depends on the magnitude of the vaccination rate. If vaccine supplies are low and immune responses poor, then sharing only a very small fraction of the vaccine supply is epidemiologically beneficial in terms of decreasing the overall burden. For stronger immune responses, augmenting vaccine sharing rates becomes increasingly beneficial from an epidemiological perspective, as the protective effects of the vaccine are maintained for longer within the population (compare the columns of the middle row of Fig. 2A). Similarly, as vaccine supplies increase (compare the colored curves in the middle row of Fig. 2A), the minimum value of infections occurs for increasingly large values of *f*, or fractions of vaccines shared. Eventually, when global vaccination rates are high, even for poor host immune responses, this minimum is attained when more than half of the vaccine supply is allocated to the LAR (leftmost panel of middle row of Fig. 2A). By symmetry, the opposite occurs if there is less transmission in the LAR. These trends are further magnified if overall transmission rates are increased (Fig. 2B). To further emphasize these effects, we present the long-term equilibrium of each country under representative scenarios in Fig. 2, C to E. In particular, the comparison between Fig. 2, D and E, illustrates the importance, and indirect benefit, of increasing vaccine supply. The relative sizes of the HAR and LAR populations can have important consequences for the fraction of vaccine allocation that minimizes the weighted fraction of infections (figs. S1 and S2). Overall, these results highlight the importance of continued NPIs that decrease transmission, such as rapid-testing and physical distancing, in conjunction with ramping up vaccination and sharing vaccine supplies equitably to decrease overall burden.

### Medium-term dynamics

#### Epidemiological considerations

**Fig. 3:**
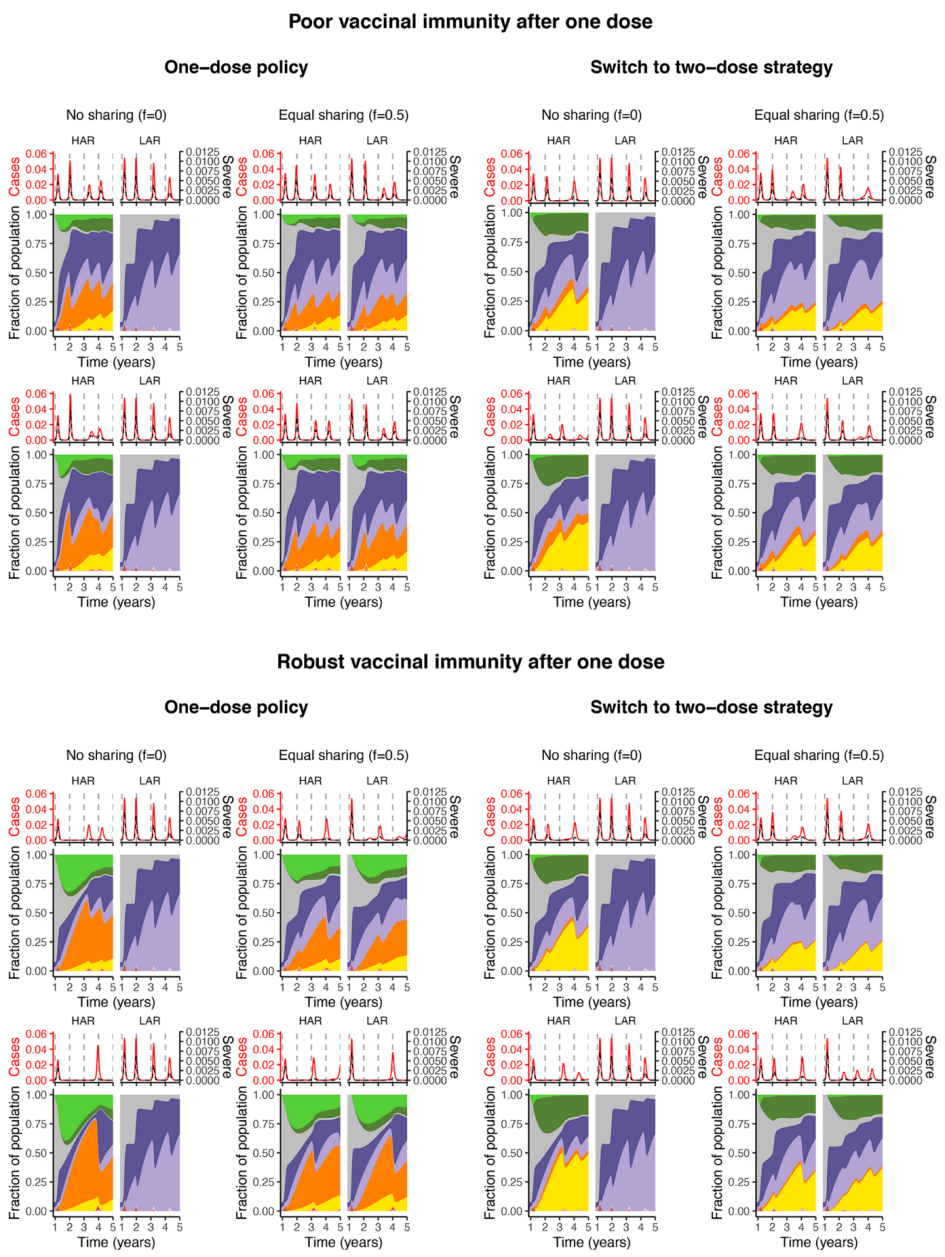
Immune landscapes and infections in both countries under a range of vaccination strategies and assumptions related to robustness of immune responses. Note that the color scheme is as in Fig. 1. In all panels, vaccination begins after week 48. *Poor vaccinal immunity after one dose* is represented by $\frac{1}{\rho_{1}}=0.25\text{ year}$ and $\epsilon_{1}=0.9$, whereas *robust vaccinal immunity after one dose* means $\frac{1}{\rho_{1}}=1\text{ year}$ and $\epsilon_{1}=0.7$. Other immunity parameter values are as follows: $\frac{1}{\delta }=\text{ 1 year}$, $\frac{1}{\rho_{2}}=1\text{ year}$, $\epsilon =\epsilon_{2}=0.7$, $\epsilon_{V_{1}}=0.1$, and $\epsilon_{V_{2}}=0.05$. All other parameters including the procedure for the calculation of severe cases are described in the supplementary materials. In both the top and bottom panels: the top row depicts a switch from a maximum first-dose administration rate of 1% to 3% after week 60, whereas it is 1% to 5% for the bottom row (and concurrent with sharing, if it occurs).

To consider the near- and medium-term dynamic epidemiological effects of vaccine sharing, in Fig. 3 we explore the landscapes of immunity and infections across multiple scenarios for otherwise-symmetric countries (i.e., population size and seasonal transmission rates). In all scenarios, vaccine supply is assumed to be limited initially in the HAR [modeled as a one dose policy, a lower maximal rate of administration of the first dose $\nu_{0,\text{tot}}$, and no sharing (*f* = 0)] and then is assumed to increase. In conjunction with an increase in $\nu_{0,\text{tot}}$, we allow for a transition to the recommended two-dose strategy ($\frac{1}{\omega }=4\text{ weeks}$, right two columns of Fig. 3), and/or the initiation of equal sharing (*f* = 0.5) with the LAR (which is assumed to distribute vaccines using the same strategy as the HAR) may be initiated (second and fourth columns of Fig. 3).

Intuitively, if one-dose immunity is robust (bottom panel of Fig. 3), then transitioning to a two-dose strategy leads to fewer individuals with robust vaccinal immunity, in turn giving rise to increases in infections in both in the short- and medium-term [compare the corresponding scenarios of the bottom panel of Fig. 3 and see also (*6*)]. In such a situation, ‘one-dose’ strategies (i.e. either the first dose of a 2-dose vaccine or the unique dose of a 1-dose vaccine) with equal sharing between countries suppress overall burden. On the other hand, if one-dose immunity is poor, switching to the recommended two-dose regimen prevents the accumulation of individuals with waned one-dose immunity and thus potentially larger infections peaks in the longer term (top panel of Fig. 3). If poor one-dose immunity nevertheless reduces severity of infection after waning (unlike our pessimistic assumption), then the predicted clinical burden of severe cases would likely be lower. Finally, if one-dose immunity is poor and a one-dose policy is pursued, the first infection peak after ramping up vaccination in the HAR may be higher without sharing (top left panel of Fig. 3). This counter-intuitive finding arises due to the large accumulation of individuals with waned one-dose immunity who experience infection. This highlights the important role for population-level susceptibility (modulated by natural and vaccinal immune responses) and its dynamical interplay with transmission in determining the timing and burden of infections. The effects of different NPI scenarios, transmission patterns, and vaccination rates in either the HAR or LAR can be further explored with the online application (*30*).

#### Evolutionary considerations

The accumulation of individuals with various states of immunity (i.e., waned one-dose immunity or immunity following natural infection) may also lead to different evolutionary outcomes depending on the vaccine sharing scheme pursued. Current evidence suggests that adaptive immune responses following natural infection with SARS-CoV-2 are fairly robust and long-lasting (*31*, *32*). This protection may be less certain in the context of subsequent infection with variant strains (*33*, *34*). Encouragingly, studies indicate that previously-infected hosts are also largely protected (clinically and against breakthrough infections) against emerging variants after a single vaccine dose (*35*). However, recent work indicates poorer protection against the rapidly spreading ‘delta’ variant of concern particularly after a single dose (*36*), although protection against severe disease still appears fairly robust (*37*–*39*). Interestingly, the protection conferred by a single vaccine dose in previously infected individuals may be even greater than that provided by two vaccine doses in naÃ¯ve hosts (*40*). However, there are still many immunological uncertainties, e.g., the duration and longer-term strength of this protection against existing strains and potential emerging variants remain unknown.

In fig. S3, we use the evolutionary framework from (*6*) to project the potential net viral adaptation rate (see supplementary materials for details and online application for additional scenarios). Overall, we find that uncertainties in evolutionary outcomes dominate our projections, echoing previous findings (*6*) (fig. S3 and online application). However, if the evolutionary potential for immune escape is highest among infections in hosts with natural immunity, the framework predicts that sharing vaccines always decreases global evolutionary potential (green curves of fig. S3) in the decoupled framework. Overall, when immunity after a single vaccine dose is robust, natural and vaccine-derived immunity will limit damaging pathogen evolution relative to the scenario with poor single dose immunity (compare the top and bottom panels of fig. S3). However, if immunity is partial or waning, ongoing transmission might accelerate adaption, supporting the need for continued monitoring of variants and their interaction with natural and vaccine-derived immunity.

Another intuitive result is that, in the LAR, sharing vaccines leads to increases in population immunity (for any dosing regime) and thus a decrease in infections and burden in the short term, even with poor one-dose immunity. In general, in the HAR, sharing decreases population immunity and increases infections in the short term. However, these changes are minimal and likely acceptable given the combined decrease in infections, illustrating the long term benefits of vaccine sharing. In particular, while local cases in the HAR may increase in the short-term, the longer-term disease risk would be lowered by sharing due to a decrease in the potential for the evolution of more transmissible or immune-escape viral variants.

## Coupled framework

So far, we have assumed that the countries have decoupled disease dynamics. This simplification for tractability ignores infection importation as well as the possible emergence of variants from regions with more persistent infections. The issues that arise from the global circulation of SARS-CoV-2, particularly the variants of concern, are of considerable public health importance. Thus, we next explore these effects using the coupled framework presented in the bottom panel of Fig. 1.

### Cumulative cases and pathogen evolution in the medium-term

**Fig. 4:**
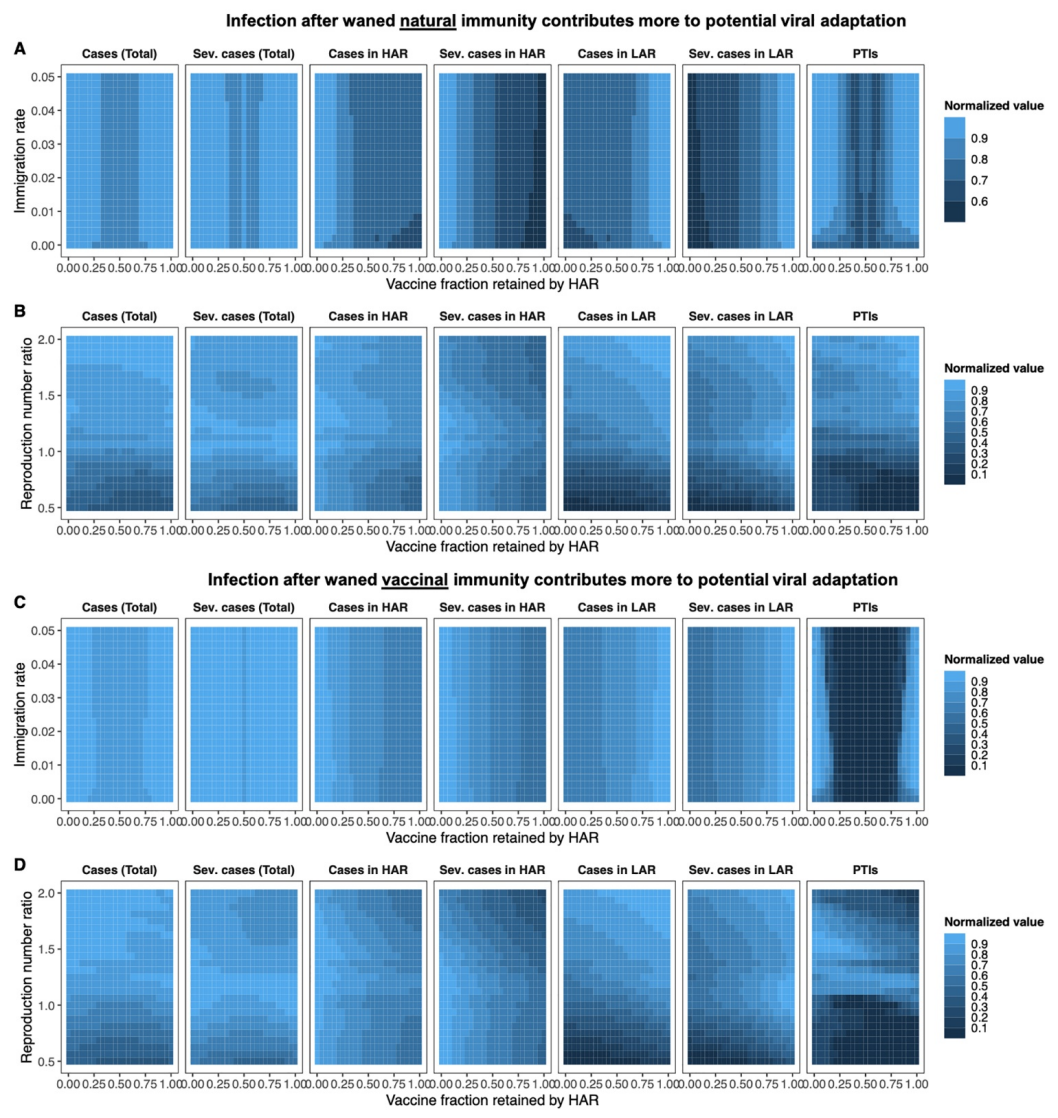
Cumulative number of cases and PTIs in the medium term. (**A** to **D**) Heat maps depicting total and severe cases from the time of vaccine onset ($t_{\text{vax}}=48\text{ weeks}$) through the end of the 5 year period for both countries (leftmost two columns), the HAR (third and fourth columns from the left), the LAR (fifth and sixth columns from the left), as well as the combined number of PTIs to have occurred in both countries at the end of 5 years (rightmost column). Each grid-point denotes the mean value of 100 simulations. The population of both countries is taken to be the same. Each area plot is internally normalized, such that the largest value in each plot is 1. The *x*-axis indicates the fraction of vaccines retained by the HAR (i.e., 1 − *f*); thus the far right of a plot is the scenario where the HAR retains all vaccines (*f* = 0). In (A) and (C), both countries have the same average transmission rate (${\bar{R}}_{0}$, see Materials and methods), and the immigration rate η is varied. In (B) and (D), the immigration rate is fixed at η = 0.01, and the relative mean transmission rate in the LAR, i.e., ${\bar{R}}_{0,\text{LAR}}/{\bar{R}}_{0,\text{HAR}}$, is varied between 0.5 and 2. The seasonality of the transmission rates in both countries and periods of NPI adoption are identical and as described in the Materials and methods. In all simulations, we assume a two-dose strategy throughout, i.e., $\frac{1}{\omega }=4\text{ weeks}$, and take the maximal rate of administration of the first dose to be $\nu_{0,\text{tot}}=2\text{\%}$. Assumed immunological parameters are $\frac{1}{\delta }=1\text{ year}$, ϵ=0.7, $\epsilon_{V_{1}}=0.1$, $\epsilon_{V_{2}}=0.05$, $\epsilon_{2}=0.7$, $\frac{1}{\rho_{2}}=1\text{ year}$, and the one- to two-dose immune response ratio is $x_{e}=0.8$ (see Materials and methods). In the top panel [(A) and (B)], we assume that infection after waned natural immunity contributes more to potential viral adaptation, and take $w_{\text{IS}}=0.8$, $w_{\text{IS}1}=0.2/x_{e}$, and $w_{\text{IS}2}=0.2$ (see Materials and methods). In the bottom panel [(C) and (D)], we assume that infection after waned vaccinal immunity contributes more to potential viral adaptation, and take $w_{\text{IS}}=0.4$, $w_{\text{IS}1}=0.8$, and $w_{\text{IS}2}=0.8\times x_{e}$ (see Materials and methods). Additional details related to the determination of severe cases are also provided in the supplementary materials.

In Fig. 4, we plot the cumulative number of total and severe cases (see supplementary materials for details) assuming equal population sizes in both countries from the time of vaccine introduction until 5 years after the pandemic onset in the HAR, LAR, and combined, as well as the projected number of PTIs to have occurred in both regions by the end of the 5 year period. We do so for various vaccine allocation fractions between the HAR and LAR, as well as a range of immigration rates assuming symmetric transmission rates (Fig. 4, A and C) and relative mean reproduction numbers assuming constant immigration rates (Fig. 4, B and D; see Materials and methods). In Fig. 4, A and B, we assume that infection following waned natural immunity contributes the most to viral adaptation, while in Fig. 4, C and D, we assume that infection following waned vaccinal immunity, and one-dose immunity in particular, contributes the most.

#### Dependence on immigration rate

For equal population sizes and symmetric transmission rates, a weak dependence of total and severe cases as well as PTIs on the immigration rate η is observed, regardless of whether infection after waned natural or vaccinal immunity is assumed to contribute more to viral evolution (Fig. 4, A and C). Additionally, more equitable vaccine distribution minimizes total PTIs and combined cases in both scenarios. When natural infections contribute more to evolution (Fig. 4A) and η is low, the HAR must retain an increasing share of the vaccines to minimize local total cases as the immigration rate increases, but this is done at the expense of more cases in the LAR and PTIs. Notably, for *f* ≈ 0, severe cases are minimized regardless of the immigration rate in the HAR and maximized in the LAR, which may have important clinical consequences. When infection following waned vaccinal immunity contributes more to viral evolution, this approach is no longer advantageous for the HAR, and the retained vaccine fraction sets the observed case numbers nearly independently of the assumed immigration rate. Further, large asymmetries in vaccine sharing (i.e., *f* = 0 or *f* = 1) result in much more marked relative numbers of PTIs in this scenario.

For the same total vaccine availability, the most realistic population asymmetry is that the LAR has a larger population. Under this condition (fig. S4) and when infection after waned natural immunity contributes more to evolution, total cases in the LAR are relatively insensitive to the amount of vaccine allocated, except for very large *f*; however severe cases can be substantially reduced with vaccine sharing. Here, combined total cases and PTIs are minimized by minimizing cases in the HAR. When a greater number of total vaccines are available (i.e., a larger $\nu_{0,\text{tot}}$; fig. S5), total and severe cases in the more populous LAR decrease approximately monotonically with increasing vaccine allocation *f* and more equitable vaccine allocation once again minimizes combined total and severe cases. When infection after waned vaccinal immunity contributes more to evolution, the trends are more similar to those for symmetric population sizes, and more equitable vaccine sharing is favored for both vaccination rates $\nu_{0,\text{tot}}$ given a larger population in the LAR (figs. S4 and S5).

When the LAR has a smaller population (fig. S6), a relatively weak dependence of total and severe case numbers and PTIs on η is still observed for both evolutionary scenarios, particularly for higher immigration rates. However, and particularly when infection after waned natural immunity contributes more to evolution, the minima in combined cases and PTIs are now observed for *f* < 0.5, i.e., when the HAR retains more than half of the available vaccines. Importantly, we note that additional booster doses may further change these landscapes of immunity, and consequently the projected burdens of total and severe cases.

#### Dependence on relative transmission rate

The number of total and severe cases and PTIs show a greater sensitivity to the average reproduction number ratio ${\bar{R}}_{0,\text{LAR}}/{\bar{R}}_{0,\text{HAR}}$ between the two countries for a fixed immigration rate. Intuitively, for equal population sizes (Fig. 4) and when ${\bar{R}}_{0}$ in the LAR country is lower, having the HAR retain more than half of the vaccines (*f* < 0.5) is a good strategy for minimizing total PTIs and cases, regardless of the evolutionary scenario. Indeed, the optimal vaccine allocation shifts closer and closer to equal sharing as the ${\bar{R}}_{0}$ values of both countries approach each other, along with an increase in cases. These trends are similar when the LAR has a larger or smaller population than the HAR (figs. S4 and S6, respectively).

When ${\bar{R}}_{0}$ in the LAR is higher, trends are more complex. In general, regardless of the relative population size or evolutionary scenario, for a given ${\bar{R}}_{\text{0,LAR}}/{\bar{R}}_{\text{0,HAR}}$, cases in the HAR decrease with increasing vaccine retention (smaller *f*), while cases in the LAR increase (Fig. 4 and figs. S4 to S6). Severe cases in each region in particular are strongly reduced by increased vaccine availability. The increase in cases in the LAR is increasingly large at higher ${\bar{R}}_{0,\text{LAR}}/{\bar{R}}_{0,\text{HAR}}$. When infection after waned natural immunity contributes more to evolution and vaccine supply is sufficiently high to reduce case numbers in the LAR, PTIs are numerous when the HAR retains a large fraction of the vaccines (Fig. 4B and figs. S5B and S6B). This is due to sustained elevated case numbers in unvaccinated individuals in the LAR. On the other hand, when infection following waned vaccinal immunity contributes more to evolution, then having the HAR (with lower transmission rates) retain a larger fraction of the vaccines minimizes PTIs for any relative population size, since the high ${\bar{R}}_{0}$ in the LAR would result in large subsequent peaks containing individuals whose vaccinal immunity has waned with sharing (Fig. 4D and figs. S4D, S5D, and S6D). However, this strategy also leads to highly elevated case numbers, including severe cases, in the LAR. Since LARs may also have more fragile healthcare systems, the elevated clinical burden under this scenario may be particularly problematic.

### Comparison of coupled and decoupled frameworks

**Fig. 5:**
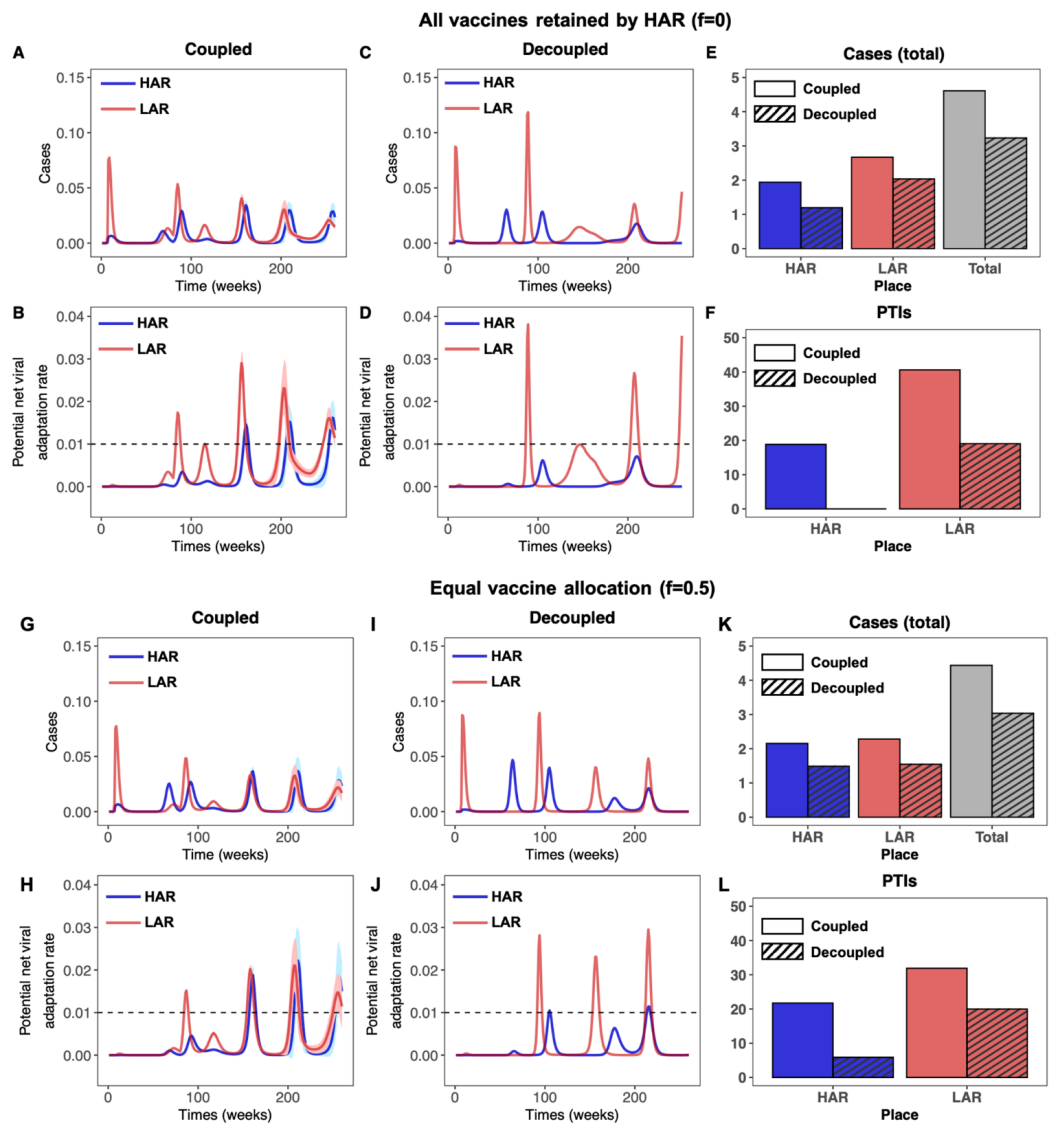
Time series of cases and potential net viral adaptation rates. (**A** to **F**) Infections in the HAR (blue) and LAR (red) for the first 5 years after pandemic onset for the coupled (left) and decoupled (middle) frameworks. Each simulation is run 100 times, with the average indicated by the solid line and the standard deviation shown with the corresponding ribbon. The average number of cumulative cases over all simulations from the time of vaccine onset $t_{\text{vax}}=48\text{ weeks}$ through the end of the 5 year period are shown in the rightmost figure for the HAR, LAR, and both countries combined for the coupled (solid) and decoupled (dashed) frameworks. (**G** to **L**) Time series of the potential viral adaptation rate in both regions for the coupled (left) and decoupled (right) frameworks. The colors, averages and standard deviations are as described above. The dashed horizontal line denotes $e_{\text{cutoff}}=0.01$, the assumed threshold for the occurrence of a PTI (see Materials and methods). The average number of PTIs at the end of the 5 year period are shown in the rightmost figure for the HAR and LAR for the coupled (solid) and decoupled (dashed) frameworks. The top panel [(A) to (F)] corresponds to the HAR retaining all vaccines (*f* = 0), while the bottom panel [(G) to (L)] corresponds to equal vaccine sharing (*f* = 0.5). In all simulations, we take ${\bar{R}}_{0,\text{LAR}}/{\bar{R}}_{0,\text{HAR}}=1.2$, η = 0.01, and assume that infection after waned natural immunity contributes primarily to evolution (i.e., $w_{\text{IS}}=0.8$, $w_{\text{IS}1}=0.2/x_{e}$, and $w_{\text{IS}2}=0.2$). All other parameters are identical to those in Fig. 4.

We note that assumptions of large ${\bar{R}}_{0}$ in the LAR also result in very large initial infection peaks, which increase community immunity in the medium-term. These initial waves are not reflected in the total case counts, however, since these values are summations from the time of vaccine initiation through the end of the 5 year period after the onset of the pandemic. Further, in Fig. 5 we illustrate the temporal effect of the coupled framework on the infection dynamics in the LAR and HAR relative to the first model with no immigration or explicit effect of PTIs on the transmission rate. When moderate asymmetry in ${\bar{R}}_{0}$ is assumed (${\bar{R}}_{0,\text{LAR}}/{\bar{R}}_{0,\text{HAR}}=1.2$), simulations using the decoupled framework suggest that a strategy where the HAR retains all vaccines would be highly beneficial for that country (top panel of Fig. 5; no PTIs are projected to occur, and low case numbers are observed throughout). However, this occurs at the expense of PTIs and infection burden in the LAR, which are both substantially higher. With the more realistic coupled framework, immigration and increases in transmission illustrate that this strategy is far less beneficial to the HAR than the decoupled framework would suggest, as substantially higher case numbers and pathogen evolution are predicted in this region. Although total cases in the HAR increase slightly when vaccines are equally distributed under the coupled framework (lower panel of Fig. 5), substantial reductions in case numbers in the LAR result in fewer PTIs in that country, and total combined case numbers are also slightly lower. To untangle the effects of immigration and PTIs on dynamics in the coupled framework, we reproduce Figs. 4 and 5 allowing for immigration only in figs. S7 and S8, respectively. In other words, these figures represent a more optimistic evolutionary scenario in which the occurrence of a PTI does not increase transmission rates in either the HAR or LAR. Overall, we show that vaccines play an important role in minimizing cases (particularly severe cases) as well as potential viral adaptation in both regions. We also emphasize that imperfect vaccinal and natural immunity and asymmetries in population size and transmission rates add many nuances to this picture.

## Caveats

A full list of caveats and future directions is presented in the supplementary materials; we briefly summarize them below. First, building on prior work and in order to focus on qualitative features, we ignore heterogeneities within countries, such as due to age (*41*) or superspreading (*42*). We have also assumed simple scenarios for nonpharmaceutical interventions as in (*6*), and assumed that the seasonal transmission rates are similar in both countries. More granular, well-parameterized epidemiological models with these complexities would lead to more accurate quantitative predictions. Additionally, underlying differences in susceptibility between the HAR and LAR, through factors such as variations in age structure or degrees of pre-existing population-level immunity, may result in differential rates of severe cases and hospitalizations independent of vaccination. An explicit treatment of age-structure and serostatus [as in (*21*)] in modeling vaccine roll-out to estimate clinical burdens in HARs and LARs under different sharing schemes should be investigated further. We also note that our models focused on the situation where dose-administration is contingent on supply, so that ‘sharing’ vaccines from the HAR to the LAR decreases the vaccination rate in the HAR. However, certain HARs may have access to more doses than they can administer. In such a scenario, sharing would be even more beneficial: it would lead to no change in the HAR vaccination rate while increasing that of the LAR.

Moving forward, additional booster doses may be administered that could alter population-level immune landscapes, and including these in future models will be important for qualitative and quantitative predictions. Additionally, we omit vaccine hesitancy (*23*), though simple extensions of our previous models with hesitancy (*5*, *6*) could examine the resulting interplay with vaccine nationalism. Furthermore, our equilibrium analyses highlight the long-term importance of vaccine sharing regardless of when sharing is initiated. However, our scenarios that examine short- and medium-term implications strongly emphasize the benefits of close to “parallel” or concurrent vaccine sharing. The effects of delaying the initiation of sharing, say until a certain proportion of individuals in the HAR are vaccinated, should be carefully examined.

Lastly, we have assumed the simplest evolutionary models, both for determining potential viral adaptation rates as well as for simulating potential increases in transmission rates. As more data become available, these should be refined (*43*–*46*), with possible directions including extending the model to explicitly track the transmission of different strains, and accounting for potential reductions in the strength of vaccinal immunity (i.e., the parameters $\epsilon_{V_{1}}$, $\epsilon_{V_{2}}$, $\epsilon_{1}$, and $\epsilon_{2}$) due to the emergence of novel immune escape variants (*47*). In particular, prolonged infections in immunosuppressed hosts may be disproportionately important for evolution (*48*). The online interactive application (*30*) allows for an in-depth exploration of the effect of different climate-driven seasonal transmission rates as well as a broad range of assumptions related to NPIs and immuno-epidemiological parameters.

## Conclusion

Even as vaccine production increases, a number of countries are choosing to share little or no vaccines with countries that have very low vaccine availability. Vaccine nationalism, dosing regimes, and host immune responses have important interactive effects, and these will substantially shape epidemiological dynamics and evolutionary potential in the medium term. Additionally, unstable vaccine supply will also increase variability in the timing or availability of first and second doses.

Using extensions of our prior work (*5*, *6*), we incorporated vaccine sharing scenarios in two countries whose infection dynamics are either otherwise independent or coupled through immigration of infectious individuals and evolution-driven increases in transmission rates. When country profiles are symmetric, we find that sharing vaccines with countries that have low availability decreases overall infections and may also mitigate potential antigenic evolution. Asymmetries in population size or transmission rates introduce additional complexities, which are particularly marked when natural and vaccinal immunity is weak. Nevertheless, our models indicate that the prompt redistribution of vaccine surpluses is likely advantageous in terms of epidemiological and evolutionary outcomes in both countries and, by extension, globally. Ethical arguments also support this policy (*13*, *14*). Persistent elevated disease transmission in countries with low vaccine availability also substantially undermines attempts at infection control via stockpiling in the country with high vaccine availability, which is not accounted for when disease transmission in both countries is assumed to be decoupled. Overall, our work highlights the importance of continued efforts in quantifying the robustness of immunity following vaccination. Furthermore, reevaluation of stockpiling policies as vaccine supplies increase is imperative, and ramping up global vaccination efforts is crucial.

## Materials and methods

### Determination of seasonal reproduction numbers

In order to reflect observed seasonal variation in transmission rates for respiratory infections arising from related coronaviruses (*49*), influenza (*49*) and respiratory syncytial virus (*50*), we base seasonal reproduction numbers in this work on those in (*5*), which were calculated in (*49*) based on the climate of New York City. Other seasonal patterns can be explored using the interactive online application. In all simulations, we modify these values to force a mean value for the basic reproduction number of ${\bar{R}}_{0}=\langle R_{0}\left(t\right)\rangle =2.3$ by multiplying the climate-derived time series $R_{0,c}\left(t\right)$ by 2.3 and dividing by its average value, i.e.,


$$R_{0}\left(t\right)=R_{0,c}\left(t\right)\frac{2.3}{{\bar{R}}_{0,c}}$$

When transmission rates are assumed to be asymmetrical, the mean value of the $R_{0}\left(t\right)$ time series is adjusted by the desired relative ratio. For example, for ${\bar{R}}_{0,\text{LAR}}/{\bar{R}}_{0,\text{HAR}}=1.2$, the reproduction number time series in the HAR is as above, but in the LAR it is


$$R_{0,\text{LAR}}\left(t\right)=R_{0,c}\left(t\right)\frac{2.3}{{\bar{R}}_{0,c}}\frac{{\bar{R}}_{0,\text{LAR}}}{{\bar{R}}_{0,\text{HAR}}}=R_{0,c}\left(t\right)\frac{2.3}{{\bar{R}}_{0,c}}\times 1.2$$

### Modeling of nonpharmaceutical interventions (NPIs)

In all simulations, we enforce periods of NPI adoption (arising from behaviors and policies such as lock downs, mask-wearing, and social distancing) in which the transmission rate is reduced from its seasonal value described in the previous section.

For the simulations of medium-term dynamics using the decoupled framework (i.e., Fig. 3 and fig. S3), we used the same NPI scenarios as in (*6*). For all simulations using the coupled framework (i.e., Figs. 4 and 5 and figs. S4 to S8), we assume that NPIs are adopted between weeks 8 and 44 following the pandemic onset resulting in the transmission rate being reduced to 40% of its seasonal value. Between weeks 45 and 79, we assume that the transmission rate is 60% of its seasonal value; higher than during the previous period due to either behavioral changes following the introduction of the vaccine or the emergence of more transmissible strains. Finally, we assume that NPIs are completely relaxed beyond week 80.

### Simple vaccination model

The complete mathematical details relevant to the following components of the simple vaccination model are presented in the supplementary materials:

Model equations

Equilibrium calculations

### Model with explicit dosing regimes

The complete mathematical details relevant to the following components of the model with explicit dosing regimes are presented in the supplementary materials:

Model equations

Calculation of cumulative case numbers

Linking vaccination rate to inter-dose period

Determination of potential net viral adaptation rate

Specification of one-dose vaccine efficacy from one- to two-dose immune response ratio

Calculation of the number of severe cases

Details for coupling between the countries

## Supplementary Materials

science.sciencemag.org/cgi/content/full/science.abj7364/DC1
Supplementary Text Figs. S1 to S8 References (*52*–*67*) MDAR Reproducibility Checklist

## References

[1] E. Dong, H. Du, L. Gardner, An interactive web-based dashboard to track COVID-19 in real time. Lancet Infect. Dis. 20, 533–534 (2020). 10.1016/S1473-3099(20)30120-1 32087114 PMC7159018

[2] F. P. Polack, S. J. Thomas, N. Kitchin, J. Absalon, A. Gurtman, S. Lockhart, J. L. Perez, G. Pérez Marc, E. D. Moreira, C. Zerbini, R. Bailey, K. A. Swanson, S. Roychoudhury, K. Koury, P. Li, W. V. Kalina, D. Cooper, R. W. Frenck Jr., L. L. Hammitt, Ö. Türeci, H. Nell, A. Schaefer, S. Ünal, D. B. Tresnan, S. Mather, P. R. Dormitzer, U. Şahin, K. U. Jansen, W. C. Gruber, C4591001 Clinical Trial Group, Safety and efficacy of the BNT162b2 mRNA covid-19 vaccine. N. Engl. J. Med. 383, 2603–2615 (2020). 10.1056/NEJMoa2034577 33301246 PMC7745181

[3] M. Voysey, S. A. C. Clemens, S. A. Madhi, L. Y. Weckx, P. M. Folegatti, P. K. Aley, B. Angus, V. L. Baillie, S. L. Barnabas, Q. E. Bhorat, S. Bibi, C. Briner, P. Cicconi, A. M. Collins, R. Colin-Jones, C. L. Cutland, T. C. Darton, K. Dheda, C. J. A. Duncan, K. R. W. Emary, K. J. Ewer, L. Fairlie, S. N. Faust, S. Feng, D. M. Ferreira, A. Finn, A. L. Goodman, C. M. Green, C. A. Green, P. T. Heath, C. Hill, H. Hill, I. Hirsch, S. H. C. Hodgson, A. Izu, S. Jackson, D. Jenkin, C. C. D. Joe, S. Kerridge, A. Koen, G. Kwatra, R. Lazarus, A. M. Lawrie, A. Lelliott, V. Libri, P. J. Lillie, R. Mallory, A. V. A. Mendes, E. P. Milan, A. M. Minassian, A. McGregor, H. Morrison, Y. F. Mujadidi, A. Nana, P. J. O’Reilly, S. D. Padayachee, A. Pittella, E. Plested, K. M. Pollock, M. N. Ramasamy, S. Rhead, A. V. Schwarzbold, N. Singh, A. Smith, R. Song, M. D. Snape, E. Sprinz, R. K. Sutherland, R. Tarrant, E. C. Thomson, M. E. Török, M. Toshner, D. P. J. Turner, J. Vekemans, T. L. Villafana, M. E. E. Watson, C. J. Williams, A. D. Douglas, A. V. S. Hill, T. Lambe, S. C. Gilbert, A. J. Pollard, Oxford COVID Vaccine Trial Group, Safety and efficacy of the ChAdOx1 nCoV-19 vaccine (AZD1222) against SARS-CoV-2: An interim analysis of four randomised controlled trials in Brazil, South Africa, and the UK. Lancet 397, 99–111 (2021). 10.1016/S0140-6736(20)32661-1 33306989 PMC7723445

[4] L. R. Baden, H. M. El Sahly, B. Essink, K. Kotloff, S. Frey, R. Novak, D. Diemert, S. A. Spector, N. Rouphael, C. B. Creech, J. McGettigan, S. Khetan, N. Segall, J. Solis, A. Brosz, C. Fierro, H. Schwartz, K. Neuzil, L. Corey, P. Gilbert, H. Janes, D. Follmann, M. Marovich, J. Mascola, L. Polakowski, J. Ledgerwood, B. S. Graham, H. Bennett, R. Pajon, C. Knightly, B. Leav, W. Deng, H. Zhou, S. Han, M. Ivarsson, J. Miller, T. Zaks, COVE Study Group, Efficacy and safety of the mRNA-1273 SARS-CoV-2 vaccine. N. Engl. J. Med. 384, 403–416 (2021). 10.1056/NEJMoa2035389 33378609 PMC7787219

[5] C. M. Saad-Roy, C. E. Wagner, R. E. Baker, S. E. Morris, J. Farrar, A. L. Graham, S. A. Levin, M. J. Mina, C. J. E. Metcalf, B. T. Grenfell, Immune life history, vaccination, and the dynamics of SARS-CoV-2 over the next 5 years. Science 370, 811–818 (2020). 10.1126/science.abd7343 32958581 PMC7857410

[6] C. M. Saad-Roy, S. E. Morris, C. J. E. Metcalf, M. J. Mina, R. E. Baker, J. Farrar, E. C. Holmes, O. G. Pybus, A. L. Graham, S. A. Levin, B. T. Grenfell, C. E. Wagner, Epidemiological and evolutionary considerations of SARS-CoV-2 vaccine dosing regimes. Science 372, 363–370 (2021). 10.1126/science.abg8663 33688062 PMC8128287

[7] C. M. Saad-Roy, S. A. Levin, C. J. E. Metcalf, B. T. Grenfell, Trajectory of individual immunity and vaccination required for SARS-CoV-2 community immunity: A conceptual investigation. J. R. Soc. Interface 18, 20200683 (2021). 10.1098/rsif.2020.0683 33530857 PMC8086877

[8] Public Health England, “SARS-CoV-2 variants of concern and variants in England: Technical briefing 15” (Public Health England, 2021); https://assets.publishing.service.gov.uk/government/uploads/system/uploads/attachment_data/file/993879/Variants_of_Concern_VOC_Technical_Briefing_15.pdf?fbclid=IwAR1yXHaljjsar1h6KQEAh66pFE4zAU_qID7BewdBw823FPtrSjMeSbQp-lE.

[9] S. P. Otto, T. Day, J. Arino, C. Colijn, J. Dushoff, M. Li, S. Mechai, G. Van Domselaar, J. Wu, D. J. D. Earn, N. H. Ogden, The origins and potential future of SARS-CoV-2 variants of concern in the evolving COVID-19 pandemic. Curr. Biol. 31, R918–R929 (2021). 10.1016/j.cub.2021.06.049 34314723 PMC8220957

[10] D. P. Fidler, Vaccine nationalism’s politics. Science 369, 749–749 (2020). 10.1126/science.abe2275 32792371

[11] J. Holder, Tracking coronavirus vaccinations around the world; www.nytimes.com/interactive/2021/world/covid-vaccinations-tracker.html.

[12] L. Eaton, Covid-19: WHO warns against “vaccine nationalism” or face further virus mutations. BMJ 372, n292 (2021). 33526414 10.1136/bmj.n292

[13] E. J. Emanuel, G. Persad, A. Kern, A. Buchanan, C. Fabre, D. Halliday, J. Heath, L. Herzog, R. J. Leland, E. T. Lemango, F. Luna, M. S. McCoy, O. F. Norheim, T. Ottersen, G. O. Schaefer, K.-C. Tan, C. H. Wellman, J. Wolff, H. S. Richardson, An ethical framework for global vaccine allocation. Science 369, 1309–1312 (2020). 10.1126/science.abe2803 32883884 PMC8691258

[14] E. J. Emanuel, F. Luna, G. O. Schaefer, K. C. Tan, J. Wolff, Enhancing the WHO’s proposed framework for distributing COVID-19 vaccines among countries. Am. J. Public Health 111, 371–373 (2021). 10.2105/AJPH.2020.306098 33566663 PMC7893355

[15] L. M. Herzog, O. F. Norheim, E. J. Emanuel, M. S. McCoy, Covax must go beyond proportional allocation of covid vaccines to ensure fair and equitable access. BMJ 372, m4853 (2021). 33402340 10.1136/bmj.m4853

[16] M. J. Keeling, A. Shattock, Optimal but unequitable prophylactic distribution of vaccine. Epidemics 4, 78–85 (2012). 10.1016/j.epidem.2012.03.001 22664066 PMC3381229

[17] H. Mamani, S. E. Chick, D. Simchi-Levi, A game-theoretic model of international influenza vaccination coordination. Manage. Sci. 59, 1650–1670 (2013). 10.1287/mnsc.1120.1661

[18] M. Salathé, J. H. Jones, Dynamics and control of diseases in networks with community structure. PLOS Comput. Biol. 6, e1000736 (2010). 10.1371/journal.pcbi.1000736 20386735 PMC2851561

[19] P. Klepac, R. Laxminarayan, B. T. Grenfell, Synthesizing epidemiological and economic optima for control of immunizing infections. Proc. Natl. Acad. Sci. U.S.A. 108, 14366–14370 (2011). 10.1073/pnas.1101694108 21825129 PMC3161560

[20] P. Klepac, I. Megiddo, B. T. Grenfell, R. Laxminarayan, Self-enforcing regional vaccination agreements. J. R. Soc. Interface 13, 20150907 (2016). 10.1098/rsif.2015.0907 26790996 PMC4759795

[21] K. M. Bubar, K. Reinholt, S. M. Kissler, M. Lipsitch, S. Cobey, Y. H. Grad, D. B. Larremore, Model-informed COVID-19 vaccine prioritization strategies by age and serostatus. Science 371, 916–921 (2021). 10.1126/science.abe6959 33479118 PMC7963218

[22] N. Mulberry, P. Tupper, E. Kirwin, C. McCabe, C. Colijn, Vaccine rollout strategies: The case for vaccinating essential workers early. medRxiv 2021.02.23.21252309 [Preprint] (2021); . 10.1101/2021.02.23.21252309 PMC10021761 36962089

[23] C. E. Wagner, J. A. Prentice, C. M. Saad-Roy, L. Yang, B. T. Grenfell, S. A. Levin, R. Laxminarayan, Economic and behavioral influencers of vaccination and antimicrobial use. Front. Public Health 8, 614113 (2020). 10.3389/fpubh.2020.614113 33409264 PMC7779682

[24] J. Zahradník, S. Marciano, M. Shemesh, E. Zoler, J. Chiaravalli, B. Meyer, Y. Rudich, O. Dym, N. Elad, G. Schreiber, SARS-CoV-2 RBD in vitro evolution follows contagious mutation spread, yet generates an able infection inhibitor. bioRxiv 2021.01.06.425392 [Preprint] (2021); . 10.1101/2021.01.06.425392

[25] A. Rambaut, N. Loman, O. Pybus, W. Barclay, J. Barrett, A. Carabelli, T. Connor, T. Peacock, D. L. Robertson, E. Volz, Preliminary genomic characterisation of an emergent SARS-CoV-2 lineage in the UK defined by a novel set of spike mutations (2020); https://virological.org/t/preliminary-genomic-characterisation-of-an-emergent-sars-cov-2-lineage-in-the-uk-defined-by-a-novel-set-of-spike-mutations/563.

[26] R. Burioni, E. J. Topol, Has SARS-CoV-2 reached peak fitness? Nat. Med. (2021). 10.1038/s41591-021-01421-7 34155413

[27] N. G. Davies, P. Klepac, Y. Liu, K. Prem, M. Jit, CMMID COVID-19 working group, Age-dependent effects in the transmission and control of COVID-19 epidemics. Nat. Med. 26, 1205–1211 (2020). 10.1038/s41591-020-0962-9 32546824

[28] H. M. Korevaar, A. D. Becker, I. F. Miller, B. T. Grenfell, C. J. E. Metcalf, M. J. Mina, Quantifying the impact of US state non-pharmaceutical interventions on COVID-19 transmission. medRxiv 2020.06.30.20142877 [Preprint] (2020); . 10.1101/2020.06.30.20142877

[29] B. L. Rice, A. Annapragada, R. E. Baker, M. Bruijning, W. Dotse-Gborgbortsi, K. Mensah, I. F. Miller, N. V. Motaze, A. Raherinandrasana, M. Rajeev, J. Rakotonirina, T. Ramiadantsoa, F. Rasambainarivo, W. Yu, B. T. Grenfell, A. J. Tatem, C. J. E. Metcalf, Variation in SARS-CoV-2 outbreaks across sub-Saharan Africa. Nat. Med. 27, 447–453 (2021). 10.1038/s41591-021-01234-8 33531710 PMC8590469

[30] E. Wagner, C. M. Saad-Roy, S. E. Morris, R. E. Baker, M. J. Mina, J. Farrar, E. C. Holmes, O. G. Pybus, A. L. Graham, E. J. Emanuel, S. A. Levin, C. J. E. Metcalf, B. T. Grenfell, Vaccine nationalism and the dynamics and control of SARS-CoV-2 interactive dashboard (2021); https://grenfelllab.princeton.edu/vaccine-nationalism. 10.1126/science.abj7364 PMC9835930 34404735

[31] J. M. Dan, J. Mateus, Y. Kato, K. M. Hastie, E. D. Yu, C. E. Faliti, A. Grifoni, S. I. Ramirez, S. Haupt, A. Frazier, C. Nakao, V. Rayaprolu, S. A. Rawlings, B. Peters, F. Krammer, V. Simon, E. O. Saphire, D. M. Smith, D. Weiskopf, A. Sette, S. Crotty, Immunological memory to SARS-CoV-2 assessed for up to 8 months after infection. Science 371, eabf4063 (2021). 10.1126/science.abf4063 33408181 PMC7919858

[32] S. P. Anand, J. Prévost, M. Nayrac, G. Beaudoin-Bussières, M. Benlarbi, R. Gasser, N. Brassard, A. Laumaea, S. Y. Gong, C. Bourassa, E. Brunet-Ratnasingham, H. Medjahed, G. Gendron-Lepage, G. Goyette, L. Gokool, C. Morrisseau, P. Bégin, V. Martel-Laferrière, C. Tremblay, J. Richard, R. Bazin, R. Duerr, D. E. Kaufmann, A. Finzi, Longitudinal analysis of humoral immunity against SARS-CoV-2 Spike in convalescent individuals up to 8 months post-symptom onset. Cell Reports Medicine 2, 100290 (2021). 10.1016/j.xcrm.2021.100290 33969322 PMC8097665

[33] V. Shinde, S. Bhikha, Z. Hoosain, M. Archary, Q. Bhorat, L. Fairlie, U. Lalloo, M. S. L. Masilela, D. Moodley, S. Hanley, L. Fouche, C. Louw, M. Tameris, N. Singh, A. Goga, K. Dheda, C. Grobbelaar, G. Kruger, N. Carrim-Ganey, V. Baillie, T. de Oliveira, A. Lombard Koen, J. J. Lombaard, R. Mngqibisa, A. E. Bhorat, G. Benadé, N. Lalloo, A. Pitsi, P.-L. Vollgraaff, A. Luabeya, A. Esmail, F. G. Petrick, A. Oommen-Jose, S. Foulkes, K. Ahmed, A. Thombrayil, L. Fries, S. Cloney-Clark, M. Zhu, C. Bennett, G. Albert, E. Faust, J. S. Plested, A. Robertson, S. Neal, I. Cho, G. M. Glenn, F. Dubovsky, S. A. Madhi, 2019nCoV-501 Study Group, Efficacy of NVX-CoV2373 Covid-19 vaccine against the B.1.351 variant. N. Engl. J. Med. 384, 1899–1909 (2021). 10.1056/NEJMoa2103055 33951374 PMC8091623

[34] C. A. Prete Jr., L. F. Buss, C. M. M. Abrahim, T. Salomon, M. A. E. Crispim, M. K. Oikawa, R. Buccheri, E. Grebe, A. G. da Costa, N. A. Fraiji, M. d. P. S. S. Carvalho, N. Alexander, N. R. Faria, C. Dye, V. H. Nascimento, M. P. Busch, E. C. Sabino, Reinfection by the SARS-CoV-2 P.1 variant in blood donors in Manaus, Brazil. medRxiv 2021.05.10.21256644 [Preprint] (2021); . 10.1101/2021.05.10.21256644 PMC8817641 35123418

[35] C. J. Reynolds, C. Pade, J. M. Gibbons, D. K. Butler, A. D. Otter, K. Menacho, M. Fontana, A. Smit, J. E. Sackville-West, T. Cutino-Moguel, M. K. Maini, B. Chain, M. Noursadeghi, T. Brooks, A. Semper, C. Manisty, T. A. Treibel, J. C. Moon, A. M. Valdes, Á. McKnight, D. M. Altmann, R. Boyton, UK COVIDsortium Immune Correlates Network, UK COVIDsortium Investigators, Prior SARS-CoV-2 infection rescues B and T cell responses to variants after first vaccine dose. Science 372, eabh1282 (2021). 33931567 10.1126/science.abh1282 PMC8168614

[36] J. L. Bernal, N. Andrews, C. Gower, E. Gallagher, R. Simmons, S. Thelwall, J. Stowe, E. Tessier, N. Groves, G. Dabrera, R. Myers, C. Campbell, G. Amirthalingam, M. Edmunds, M. Zambon, K. Brown, S. Hopkins, M. Chand, M. Ramsay, Effectiveness of COVID-19 vaccines against the B.1.617.2 variant. medRxiv 2021.05.22.21257658 [Preprint] (2021); . 10.1101/2021.05.22.21257658

[37] J. Stowe, N. Andrews, C. Gower, E. Gallagher, L. Utsi, R. Simmons, S. Thelwall, E. Tessier, N. Groves, G. Dabrera, R. Myers, C. Campbell, G. Amirthalingam, M. Edmunds, M. Zambon, K. Brown, S. Hopkins, M. Chand, M. Ramsay, J. L. Bernal, Effectiveness of COVID-19 vaccines against hospital admission with the Delta (B.1.617.2) variant [Preprint] (2021); https://khub.net/documents/135939561/479607266/Effectiveness+of+COVID-19+vaccines+against+hospital+admission+with+the+Delta+%28B.1.617.2%29+variant.pdf/1c213463-3997-ed16-2a6f-14e5deb0b997?t=1623689315431.

[38] S. Nasreen, H. Chung, S. He, K. A. Brown, J. B. Gubbay, S. A. Buchan, D. B. Fell, P. C. Austin, K. L. Schwartz, M. E. Sundaram, A. Calzavara, B. Chen, M. Tadrous, K. Wilson, S. E. Wilson, J. C. Kwong, Effectiveness of COVID-19 vaccines against variants of concern, Canada. medRxiv 2021.06.28.21259420 [Preprint] (2021); . 10.1101/2021.06.28.21259420

[39] D. Lieber, “Pfizer vaccine less effective against delta infections but prevents severe illness, Israeli data show,” *Wall Street Journal*, 6 July 2021; www.wsj.com/articles/pfizers-covid-19-vaccine-is-less-effective-against-delta-variant-israeli-data-show-11625572796?fbclid=IwAR1j5TqBVHtQH7LGxyc6GQUFhsuPvvVd2C7HsBd-7kvY6p-2Gk7-FipnKm4.

[40] L. Stamatatos, J. Czartoski, Y.-H. Wan, L. J. Homad, V. Rubin, H. Glantz, M. Neradilek, E. Seydoux, M. F. Jennewein, A. J. MacCamy, J. Feng, G. Mize, S. C. De Rosa, A. Finzi, M. P. Lemos, K. W. Cohen, Z. Moodie, M. J. McElrath, A. T. McGuire, mRNA vaccination boosts cross-variant neutralizing antibodies elicited by SARS-CoV-2 infection. Science 372, 1413–1418 (2021). 10.1126/science.abg9175 33766944 PMC8139425

[41] J. S. Lavine, O. N. Bjornstad, R. Antia, Immunological characteristics govern the transition of COVID-19 to endemicity. Science 371, 741–745 (2021). 10.1126/science.abe6522 33436525 PMC7932103

[42] R. Laxminarayan, B. Wahl, S. R. Dudala, K. Gopal, C. Mohan B, S. Neelima, K. S. Jawahar Reddy, J. Radhakrishnan, J. A. Lewnard, Epidemiology and transmission dynamics of COVID-19 in two Indian states. Science 370, 691–697 (2020). 10.1126/science.abd7672 33154136 PMC7857399

[43] C. M. Saad-Roy, A. B. McDermott, B. T. Grenfell, Dynamic perspectives on the search for a universal influenza vaccine. J. Infect. Dis. 219, S46–S56 (2019). 10.1093/infdis/jiz044 30715467 PMC6452315

[44] K. Koelle, S. Cobey, B. Grenfell, M. Pascual, Epochal evolution shapes the phylodynamics of interpandemic influenza A (H3N2) in humans. Science 314, 1898–1903 (2006). 10.1126/science.1132745 17185596

[45] E. M. Volz, S. L. Kosakovsky Pond, M. J. Ward, A. J. Leigh Brown, S. D. W. Frost, Phylodynamics of infectious disease epidemics. Genetics 183, 1421–1430 (2009). 10.1534/genetics.109.106021 19797047 PMC2787429

[46] E. M. Volz, K. Koelle, T. Bedford, Viral phylodynamics. PLOS Comput. Biol. 9, e1002947 (2013). 10.1371/journal.pcbi.1002947 23555203 PMC3605911

[47] T. Kustin, N. Harel, U. Finkel, S. Perchik, S. Harari, M. Tahor, I. Caspi, R. Levy, M. Leschinsky, S. K. Dror, G. Bergerzon, H. Gadban, F. Gadban, E. Eliassian, O. Shimron, L. Saleh, H. Ben-Zvi, D. Amichay, A. Ben-Dor, D. Sagas, M. Strauss, Y. S. Avni, A. Huppert, E. Kepten, R. D. Balicer, D. Nezer, S. Ben-Shachar, A. Stern, Evidence for increased breakthrough rates of SARS-CoV-2 variants of concern in BNT162b2 mRNA vaccinated individuals. medRxiv 2021.04.06.21254882 [Preprint] (2021); . 10.1101/2021.04.06.21254882 PMC8363499 34127854

[48] B. Choi, M. C. Choudhary, J. Regan, J. A. Sparks, R. F. Padera, X. Qiu, I. H. Solomon, H.-H. Kuo, J. Boucau, K. Bowman, U. D. Adhikari, M. L. Winkler, A. A. Mueller, T. Y.-T. Hsu, M. Desjardins, L. R. Baden, B. T. Chan, B. D. Walker, M. Lichterfeld, M. Brigl, D. S. Kwon, S. Kanjilal, E. T. Richardson, A. H. Jonsson, G. Alter, A. K. Barczak, W. P. Hanage, X. G. Yu, G. D. Gaiha, M. S. Seaman, M. Cernadas, J. Z. Li, Persistence and evolution of SARS-CoV-2 in an immunocompromised host. N. Engl. J. Med. 383, 2291–2293 (2020). 10.1056/NEJMc2031364 33176080 PMC7673303

[49] R. E. Baker, W. Yang, G. A. Vecchi, C. J. E. Metcalf, B. T. Grenfell, Susceptible supply limits the role of climate in the early SARS-CoV-2 pandemic. Science 369, 315–319 (2020). 10.1126/science.abc2535 32423996 PMC7243362

[50] R. E. Baker, A. S. Mahmud, C. E. Wagner, W. Yang, V. E. Pitzer, C. Viboud, G. A. Vecchi, C. J. E. Metcalf, B. T. Grenfell, Epidemic dynamics of respiratory syncytial virus in current and future climates. Nat. Commun. 10, 5512 (2019). 10.1038/s41467-019-13562-y 31797866 PMC6892805

[51] C. E. Wagner, C. M. Saad-Roy, S. E. Morris, R. E. Baker, M. J. Mina, J. Farrar, E. C. Holmes, O. G. Pybus, A. L. Graham, E. J. Emanuel, S. A. Levin, Code for Vaccine nationalism and the dynamics and control of SARS-CoV-2, Zenodo (2021); . 10.5281/zenodo.5119582 PMC9835930 34404735

[52] R. M. Anderson, R. M. May, *Infectious Diseases of Humans: Dynamics and Control* (Oxford Univ. Press, 1992).

[53] O. Diekmann, J. A. P. Heesterbeek, J. A. J. Metz, On the definition and the computation of the basic reproduction ratio *R*_0_ in models for infectious diseases in heterogeneous populations. J. Math. Biol. 28, 365–382 (1990). 10.1007/BF00178324 2117040

[54] P. van den Driessche, J. Watmough, Reproduction numbers and sub-threshold endemic equilibria for compartmental models of disease transmission. Math. Biosci. 180, 29–48 (2002). 10.1016/S0025-5564(02)00108-6 12387915

[55] S. M. Kissler, C. Tedijanto, E. Goldstein, Y. H. Grad, M. Lipsitch, Projecting the transmission dynamics of SARS-CoV-2 through the postpandemic period. Science 368, 860–868 (2020). 10.1126/science.abb5793 32291278 PMC7164482

[56] J. Zhang, M. Litvinova, W. Wang, Y. Wang, X. Deng, X. Chen, M. Li, W. Zheng, L. Yi, X. Chen, Q. Wu, Y. Liang, X. Wang, J. Yang, K. Sun, I. M. Longini Jr., M. E. Halloran, P. Wu, B. J. Cowling, S. Merler, C. Viboud, A. Vespignani, M. Ajelli, H. Yu, Evolving epidemiology and transmission dynamics of coronavirus disease 2019 outside Hubei province, China: A descriptive and modelling study. Lancet Infect. Dis. 20, 793–802 (2020). 10.1016/S1473-3099(20)30230-9 32247326 PMC7269887

[57] D. H. Morris, V. N. Petrova, F. W. Rossine, E. Parker, B. T. Grenfell, R. A. Neher, S. A. Levin, C. A. Russell, Asynchrony between virus diversity and antibody selection limits influenza virus evolution. eLife 9, e62105 (2020). 10.7554/eLife.62105 33174838 PMC7748417

[58] Y. H. Hsieh, P. van den Driessche, L. Wang, Impact of travel between patches for spatial spread of disease. Bull. Math. Biol. 69, 1355–1375 (2007). 10.1007/s11538-006-9169-6 17318677 PMC7088731

[59] S. Funk, M. Salathé, V. A. A. Jansen, Modelling the influence of human behaviour on the spread of infectious diseases: A review. J. R. Soc. Interface 7, 1247–1256 (2010). 10.1098/rsif.2010.0142 20504800 PMC2894894

[60] E. P. Fenichel, C. Castillo-Chavez, M. G. Ceddia, G. Chowell, P. A. G. Parra, G. J. Hickling, G. Holloway, R. Horan, B. Morin, C. Perrings, M. Springborn, L. Velazquez, C. Villalobos, Adaptive human behavior in epidemiological models. Proc. Natl. Acad. Sci. U.S.A. 108, 6306–6311 (2011). 10.1073/pnas.1011250108 21444809 PMC3076845

[61] R. F. Arthur, J. H. Jones, M. H. Bonds, Y. Ram, M. W. Feldman, Adaptive social contact rates induce complex dynamics during epidemics. PLOS Comput. Biol. 17, e1008639 (2021). 10.1371/journal.pcbi.1008639 33566839 PMC7875423

[62] R. C. Tyson, S. D. Hamilton, A. S. Lo, B. O. Baumgaertner, S. M. Krone, The timing and nature of behavioural responses affect the course of an epidemic. Bull. Math. Biol. 82, 14 (2020). 10.1007/s11538-019-00684-z 31932981 PMC7223272

[63] P. C. Jentsch, M. Anand, C. T. Bauch, Prioritising COVID-19 vaccination in changing social and epidemiological landscapes: A mathematical modelling study. Lancet Infect. Dis. 21, 1097–1106 (2021). 10.1016/S1473-3099(21)00057-8 33811817 PMC8012029

[64] S. A. Pedro, F. T. Ndjomatchoua, P. Jentsch, J. M. Tchuenche, M. Anand, C. T. Bauch, Conditions for a second wave of COVID-19 due to interactions between disease dynamics and social processes. Front. Phys. 8, 574514 (2020). 10.3389/fphy.2020.574514

[65] S. Moore, E. M. Hill, M. J. Tildesley, L. Dyson, M. J. Keeling, Vaccination and non-pharmaceutical interventions for COVID-19: A mathematical modelling study. Lancet Infect. Dis. 21, 793–802 (2021). 10.1016/S1473-3099(21)00143-2 33743847 PMC7972312

[66] H. W. Hethcote, H. W. Stech, P. Van Den Driessche, Nonlinear oscillations in epidemic models. SIAM J. Appl. Math. 40, 1–9 (1981). 10.1137/0140001

[67] G. Röst, T. Tekeli, Stability and oscillations of multistage SIS models depend on the number of stages. Appl. Math. Comput. 380, 125259 (2020). 10.1016/j.amc.2020.125259

